# The Impact of Weight Loss Prior to Hospital Readmission

**DOI:** 10.3390/jcm12093074

**Published:** 2023-04-24

**Authors:** Kellie Fusco, Yogesh Sharma, Paul Hakendorf, Campbell Thompson

**Affiliations:** 1 Adelaide Medical School, Faculty of Health and Medical Sciences, The University of Adelaide, Adelaide, SA 5005, Australia; kellie.fusco@adelaide.edu.au; 2 Department of General Medicine, Division of Medicine, Cardiac & Critical Care, Flinders Medical Centre, Bedford Park, SA 5042, Australia; yogesh.sharma@sa.gov.au; 3 College of Medicine and Public Health, Flinders University, Adelaide, SA 5001, Australia; paul.hakendorf@flinders.edu.au

**Keywords:** unplanned hospital readmissions, clinical outcomes, length of stay, weight loss

## Abstract

Hospital readmissions place a burden on hospitals. Reducing the readmission number and duration will help reduce the burden. Weight loss might affect readmission risk, especially the risk of an early (<30 days) readmission. This study sought to identify the predictors and the impact of weight loss prior to a delayed readmission (>30 days). Body mass index (BMI) was measured during the index admission and first readmission. Patients, after their readmission, were assessed retrospectively to identify the characteristics of those who had lost >5% weight prior to that readmission. Length of stay (LOS), time spent in the intensive care unit (ICU) and the one-year mortality of those patients who lost weight were compared to the outcomes of those who remained weight-stable using multilevel mixed-effects regression adjusting for BMI, Charlson comorbidity index (CCI), ICU hours and relative stay index (RSI). Those who were at risk of weight loss prior to readmission were identifiable based upon their age, BMI, CCI and LOS. Of 1297 patients, 671 (51.7%) remained weight-stable and 386 (29.7%) lost weight between admissions. During their readmission, those who had lost weight had a significantly higher LOS (IRR 1.17; 95% CI 1.12, 1.22: *p* < 0.001), RSI (IRR 2.37; 95% CI 2.27, 2.47: *p* < 0.001) and an increased ICU LOS (IRR 2.80; 95% CI 2.65, 2.96: *p* < 0.001). This study indicates that weight loss prior to a delayed readmission is predictable and leads to worse outcomes during that readmission.

## 1. Introduction

The avoidance or shortening of the duration of any readmission is a target for health care improvement because these actions will improve hospital congestion if not patient outcomes. Flares in chronic conditions are frequently the cause of an unplanned hospital admission [1,2,3,4,5,6], and patients with chronic conditions are often subsequently readmitted [1]. These chronic conditions include, but are not limited to, chronic heart failure, chronic kidney disease, depression, metastatic cancer, bacterial pneumonia, chronic obstructive pulmonary disease (COPD), hypertension and stroke. In fact, these chronic illnesses not only increase the risk of readmission, but they can also be associated with weight loss. Weight loss and malnutrition have been identified as risk factors for an early readmission (within 30 days of discharge) [4,7,8,9], but there are no studies that have looked at how weight change prior to a delayed readmission (>30 days following discharge) impacts upon the outcomes of the patient during and after the readmission episode.

Unplanned hospital readmission is mostly studied for readmissions up to 30 days following discharge. There are several studies that have looked at unplanned but delayed readmissions occurring after more than 30 days following discharge [1,5,7,9,10,11,12], although none of these studies have also studied the impact of weight loss prior to readmission and how this affects patient outcomes.

Since readmission and the length of stay after readmission place a considerable burden upon already congested hospitals [13,14], we sought to determine the characteristics of those at risk of experiencing weight loss prior to any delayed readmission and to determine whether weight loss after an index admission had any influence upon the outcomes of a delayed readmission to hospital. Such information, if obtained at a time for the patient when an intervention can be initiated, might help guide and improve patient care as well as improve hospital efficiency.

Therefore, the first aim of this paper is to identify, from the characteristics and outcomes of the index admission, those patients who are most likely to lose weight prior to any delayed readmission. The second aim is to determine whether pre-readmission weight loss produces any differences in health outcomes during and following the delayed readmission. For context, the characteristics and outcomes are reported of those who gained weight prior to their delayed readmission.

## 2. Materials and Methods

Routinely collected data were accessed for patients who were admitted to three South Australian teaching hospitals (the Royal Adelaide Hospital, Flinders Medical Centre and Repatriation General Hospital) between 1 January 2015 and 31 December 2018 and who were given a score using the Malnutrition Universal Screening Tool (eMUST) screening tool [15]. The eMUST database contains information such as patient identification number, date of birth, date of hospital admission, height, weight and BMI. The eMUST data were linked to the hospital inpatient database. The following variables were added through the hospital database linkage: age, sex, Charlson comorbidity index (CCI), socioeconomic category as determined by the Index of Relative Social Disadvantage (IRSD), LOS, relative stay index (RSI) defined as LOS divided by expected LOS, admission to the intensive care unit (ICU), LOS in ICU, patients’ indigenous status and hospital readmissions within 7 days and 30 days of discharge.

Our study was focused on those who experienced a delayed readmission (i.e., readmission commencing at least 30 days following discharge from the index admission). Therefore, patients who experienced in-hospital mortality during their index admission, mortality within 30 days of index discharge or readmission within 30 days of index discharge were all excluded from the analysis.

**Figure 1:**
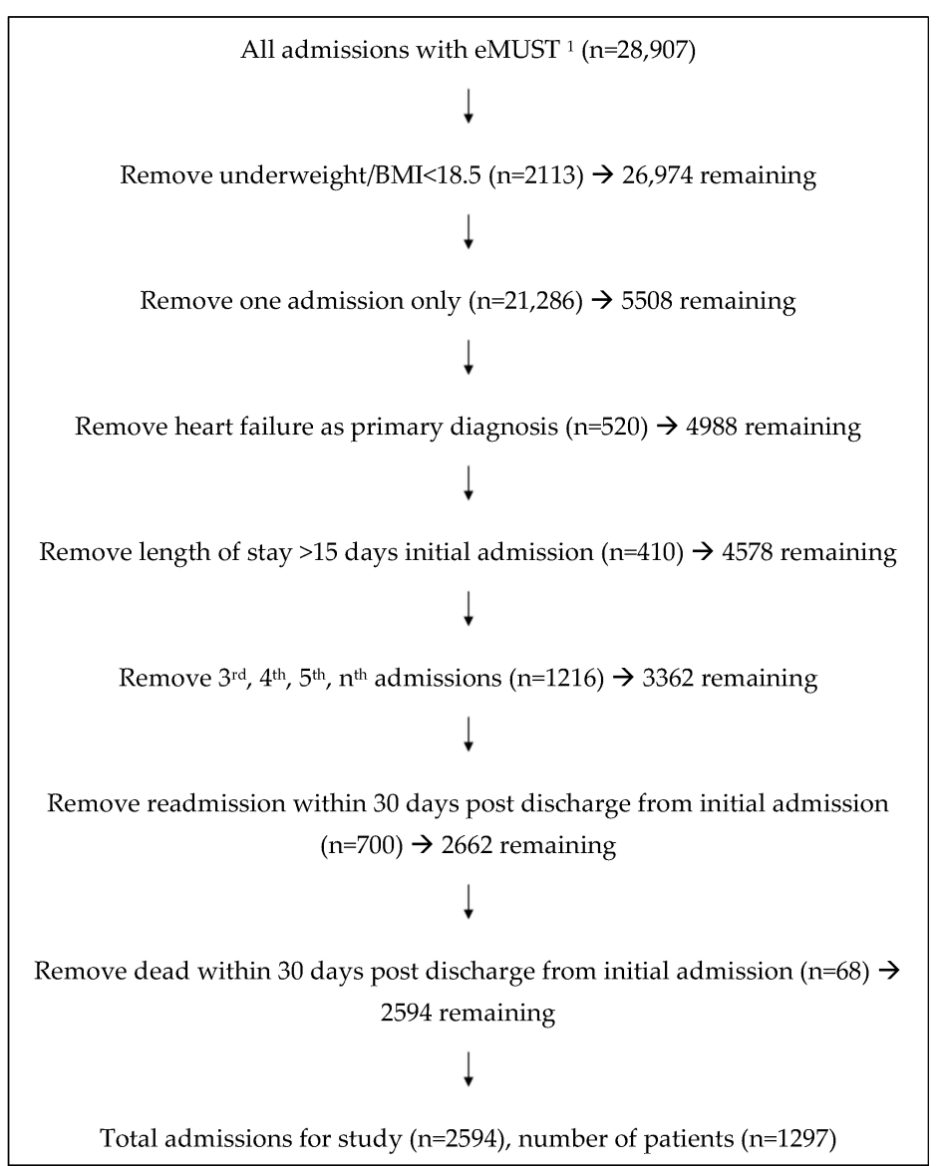
Study Flow Diagram. ^1^ eMUST is an electronic malnutrition universal screening tool which includes body mass index.

The dataset contained 28,907 admissions of patients who had an eMUST performed during their hospital admission (Figure 1). Patients with a BMI < 18.5 kg/m^2^ (n = 2113) were removed, leaving 26,974 in the dataset. All patients who were admitted only once were removed (n = 21,286), leaving 5508 index admissions and readmissions (comprising 2754 patients) in the dataset. Those with heart failure (HF) as a primary diagnosis (n = 520; 260 patients) were removed from the analysis, those with a LOS greater than 15 days for their index admission were removed (n = 410:205 patients) and subsequent admissions (third, fourth, fifth, etc.) for any patient were removed (1216 readmissions). Patients who either died within 30 days of index discharge (n = 34 patients) or were readmitted within 30 days of index discharge (n = 350 patients) were also removed from the dataset. The remaining 1297 patients (representing 2594 admissions and readmissions) were analyzed for the study.

Patients who had a BMI < 18.5 kg/m^2^ were excluded because previous evidence suggests an association of this group of patients with known health issues [16,17,18,19]. All patients with a primary diagnosis of HF were also excluded because these patients may develop significant fluid shifts manifesting as weight loss during and between hospital admissions [20,21]. Patients whose index admission LOS was greater than 15 days were excluded from the analysis due to the possibility of their weight loss occurring during a prolonged hospital stay [22,23,24].

Three categories of patients were developed based upon whether they gained weight (≥5%), lost weight (≥5%) or remained weight-stable (<5% weight change) between their first admission and their readmission. A 5% weight change was selected because it has been shown to have clinical meaning in obese patients [25]. Data were assessed for normality by a visual assessment of histograms (see supplementary files). The differences between the three weight change groups were assessed using the one-way analysis of variance (ANOVA) or the Kruskal–Wallis H test, as appropriate, for continuous variables, and the chi square test was used for the categorical variables. We used multilevel regression models to determine associations between patient characteristics/outcomes and weight change after adjustment for hospital-level variation. Similarly, the relationships between weight change and the continuous outcome variables (such as LOS and ICU hours) were examined using the mixed-effects Poisson regression model after adjustment for age, CCI, LOS during index admission, ICU time spent during index admission and the RSI during index admission, and incidence risk ratios (IRR) were determined. For categorical outcome variables (such as admission to ICU and mortality), we used the mixed-effects logistic regression model and odds ratios (OR) were determined after adjustment for the above-mentioned variables. Model specification was tested by use of the link test [26] in Stata and the linearity assumption was tested by plotting the model residuals vs. predictors using scatter graphs. To achieve proper model specification, we transformed CCI (0 = low CCI, 1 = high CCI) and BMI (18.5–24.9 kg/m^2^ = normal BMI ≥ 25 kg/m^2^ = high BMI) into categorical variables. All statistical analyses were performed by use of Stata software version 21.0 (StataCorp LP, College Station, TX, USA). All outcomes were assessed using a 2-sided type 1 error rate of alpha = 0.05.

## 3. Results

The data analyzed included 1297 patients who had an eMUST measured at both their index admission and readmission to hospital (Figure 1). The number of elapsed days from discharge to readmission for the stable weight group was 190 ((85,445) median, IQR) and for the weight loss group the median (IQR) elapsed days was 256 (103,207) and these differed significantly (*p* < 0.001).

As shown in Table 1, patients who lost weight prior to their readmission had a higher BMI (*p* = 0.036) and a higher CCI (*p* < 0.001), whilst patients who gained weight prior to their readmission were younger in age than those who were weight-stable (*p* < 0.001). There were significant differences in the outcomes of the index admission for the patients who went on to lose or gain weight prior to their readmission. Patients who lost weight prior to readmission had a longer LOS during their index admission (*p* < 0.001), higher CCI (*p* < 0.001) and spent more time in the ICU (if they were admitted to the ICU) (*p* < 0.001) when compared to weight-stable patients. The outcomes of the index admission for patients who gained weight prior to readmission indicate that these patients spent less time in the ICU (*p* = 0.001) and had a higher RSI than those who remained weight-stable.

As shown in Table 2, patients who lost weight prior to readmission had, for that readmission, a significantly increased LOS (*p* < 0.001), inpatient mortality (*p* = 0.012), one year mortality (*p* < 0.001), RSI (*p* < 0.001), number of hours in the ICU once admitted (*p* = 0.001) and were more likely to be discharged somewhere other than home (*p* < 0.001) when compared to weight-stable patients. Patients who gained weight prior to readmission had, for that readmission, a significantly increased LOS, RSI and number of hours in the ICU once admitted (all *p* < 0.001) when compared to weight-stable patients.

These outcomes of patients and their hospital admission can interact with and influence observed weight changes. Therefore, we applied a multilevel logistic regression analysis to determine the characteristics and outcomes from the index admission that were likely to result in the patient losing weight prior to their readmission. As shown in Table 3, after adjustment for age, BMI, CCI and LOS, index admission LOS remained a significant predictor for a patient to lose weight prior to readmission, as did BMI, age and higher CCI.

Table 4 shows the multilevel regression model for the outcomes of patients during their readmission to hospital. This multilevel regression model adjusts for age, BMI, CCI, LOS, ICU hours and RSI. This showed an increased LOS for patients who had lost weight since their index admission when compared to patients who remained weight-stable. These weight loss patients also had an increased RSI and increased ICU hours if admitted to the ICU. The one-year mortality was significantly increased among patients who lost weight when compared to mortality in weight-stable patients; however, there was no significant difference in the 30-day readmission rate between these two groups (Table 4).

## 4. Discussion

Of the 1297 patients who were weighed when first admitted and weighed again when readmitted to hospital during the data period, 51.7% maintained a stable weight whilst 18.5% gained weight and 29.8% lost at least 5% of their initial weight. On average, the patients recruited for this analysis were readmitted more than six months after their initial discharge and no patient readmitted within 30 days of index discharge was included.

Patients at risk of losing at least 5% of their weight prior to readmission were identifiable beforehand by their age, BMI, CCI and LOS and had worse outcomes during their readmission than those who remained weight-stable. During readmission, the patients who had lost weight had a longer LOS, RSI and LOS in the ICU than those whose weight remained stable. Patients who gained at least 5% of their weight prior to readmission were identifiable beforehand by being younger and spending less time in the ICU but they had a significantly longer RSI than those who were weight-stable. The exclusion of readmissions within 30 days of discharge ensured that any patients experiencing only immediate, short-term weight loss had been excluded from the analysis.

The predictors and outcomes of unplanned hospital readmissions have been extensively studied, but most of these readmission studies have looked at readmission within 30 days. While weight loss immediately following discharge might predict early readmission [4], studies looking at hospital readmission after 30 days have not looked at weight change between admissions [10,27,28,29]. The most relevant studies are one study which found that the significant factors for being readmitted within 60 days were (i) a cumulative inpatient LOS of more than 7 days, (ii) having a cancer diagnosis and (iii) being older than 85 years of age [27], while another relevant study found malnourished patients were at higher risk of readmission between 8 and 180 days after discharge [10].

Weight changes in the months leading up to hospital admission or leading up to unplanned hospital readmission are usually unknown. Once admitted, inpatients categorized as underweight and patients with obesity have different outcomes to those categorized as normal and overweight [9,30]. Patients with an increased BMI have been shown to have a shorter LOS, lower in-hospital mortality and improved outcomes after discharge when compared to non-obese patients [1,31], whereas underweight patients have higher LOS and mortality when compared to patients in the normal BMI range [17,18].

The present study reports markedly different outcomes for those who lost weight when compared to those who remained weight-stable. A sub-analysis looking at patients with a BMI< or >30 kg/m^2^ during their index admission did not significantly alter the present study’s findings. The current study provides no supportive evidence for counselling the obese inpatient to lose weight after discharge.

The relative risk to health of increased weight varies with age [31], and a higher percentage of readmissions occur as age increases [2,27,32]. The studies investigating age and readmission risk unfortunately varied significantly in their categorization of age groups, one looked at age over 50 years, another over 85 years. Both showed that age increased readmission risk. A third study found that age (a watershed at 85 years) did not have a significant impact on readmission [32]. A sub-analysis of our data looking at patients aged over or under 75 years at their index admission did not significantly alter the present study’s findings.

The present study did not look at the cause of the unplanned readmission but retrospectively determined the characteristics of patients that were predictive of experiencing significant weight loss before a readmission. Those readmissions occurred, on average, six months or more after discharge; all readmissions within 30 days were excluded. Patients at risk of weight loss prior to any readmission had identifiable characteristics during their index admission. Specifically, those at risk were older, had a higher BMI, a higher CCI and a longer LOS than those who were weight-stable between admissions. If patients who are not only at risk of readmission but are also at risk of weight loss prior to that readmission can be identified during their index admission, they could possibly be targeted with an intervention during or at the end of that index admission.

Over and above identifying the at-risk population during their index admission, information concerning patients’ inter-admission weight loss, available at the time of being weighed during the readmission, offers a second opportunity to highlight to clinicians a group of patients at risk of adverse health outcomes at a time when a targeted intervention is possible.

One limitation of this work is that the determination of patients’ BMIs was infrequent and only performed in approximately 10% of all admissions [30]. Admittedly, the BMI distribution of the present study matches another study of a whole hospital within Australia [33]. The present study uses retrospective data and may include unknown confounders and hence lead to incorrect inference of the results obtained. Because the present work is observational, it calls for a prospective study looking at an intervention where weight loss is prevented or avoided after discharge and prior to any readmission.

## 5. Conclusions

Those who are older, have a higher BMI, a higher CCI and a longer LOS are at greater risk of weight loss prior to a readmission. Patients who lose weight prior to a delayed (>30 days) readmission are at risk of having a worse outcome (i.e., a longer inpatient LOS and, if relevant, a longer ICU stay) during and after that readmission than if no weight loss had occurred. This prolonged LOS can adversely affect patient health as well as provoke hospital congestion issues.

## Figures and Tables

**Table 1:** Characteristics and outcomes of index admission after categorization according to weight change at subsequent admission.

Characteristics of Index Admission	Stable Weight	Weight Gain > 5%	*p* Value ^2^	Weight Loss > 5%	*p* Value ^2^	*p* Value ^3^
Total n = 1297 (%)	n = 671 (52)	n = 240 (18)		n = 386 (30)		
Age (median, IQR)	74 (58, 84)	68 (55, 81)	<0.001	74 (60, 84)	0.004	<0.001
Gender (male, n (%))	356 (53)	122 (50)	1.000	194 (50)	1.000	0.445
BMI (median, IQR)	26.5 (22.9, 30.4)	25.2 (21.5, 30.9)	0.383	26.7 (23.7, 31.4)	0.036	0.030
ATSI, n (%)	11 (2)	6 (2)	1.000	6 (2)	1.000	0.877
CCI (median, IQR)	1 (0, 2)	1 (0, 2)	0.686	1 (0, 2)	<0.001	<0.001
Outcomes of index admission						
LOS ^1^ (median, IQR)	4 (3, 8)	5 (3, 8)	0.426	5 (3, 8)	<0.001	0.002
ICU admission, n (%)	20 (3)	13 (5)	0.235	10 (3)	1.000	0.135
ICU hours (median, IQR)	45 (28.5, 74.5)	41 (17, 68)	0.001	70 (43, 87)	<0.001	<0.001
RSI median (IQR)	0.96 (0.56, 1.56)	1 (0.58, 1.67)	<0.001	0.98 (0.58, 1.60)	<0.001	<0.001

ATSI = Aboriginal/Torres Strait Islander, BMI = Body Mass Index, CCI = Charlson Comorbidity Index, LOS = length of stay, ICU = Intensive Care Unit, RSI = relative stay index, IQR = Inter-Quartile Range; ^1^ LOS adjusted for inpatient mortality, ^2^ when compared to the weight-stable group, ^3^ chi square test and ANOVA or Kruskal–Wallis H test.

**Table 2:** Outcomes of readmission after categorization according to weight change at subsequent admission.

Outcomes of Readmission	Stable Weight	Weight Gain > 5%	*p* Value ^3^	Weight Loss > 5%	*p* Value ^3^	*p* Value ^4^
LOS ^1^ median (IQR)	5 (2, 10)	6 (3, 10)	0.001	7 (3, 12)	<0.001	<0.001
Inpatient mortality, n (%)	3 (0)	5 (2)	0.188	10 (3)	0.012	0.010
One-year mortality ^2^, n (%)	67 (10)	22 (9)	1.000	69 (18)	<0.001	<0.001
RR30, n (%)	145 (22)	48 (20)	1.000	87 (23)	1.000	0.754
RSI (median, IQR)	0.95 (0.54, 1.89)	1.01 (0.54, 1.79)	<0.001	1.13 (0.60, 2.08)	<0.001	<0.001
ICU admission, n (%)	31 (5)	12 (5)	1.000	23 (6)	0.186	0.633
ICU hours (median, IQR)	59 (40, 93)	80.5 (42, 155)	<0.001	95 (63, 167)	<0.001	<0.001
Discharge elsewhere (not home), n (%)	106 (16)	44 (18)	0.068	85 (22)	0.001	0.004

RR30 = readmission within 30 days; ^1^ LOS adjusted for inpatient mortality, ^2^ excluding inpatient mortality and 30-day mortality, ^3^ when compared to the weight-stable group, ^4^ chi square test and ANOVA or Kruskal–Wallis H test.

**Table 3:** Multilevel logistic regression model comparing patient characteristics and outcomes during the index admission to predict whether weight loss will occur prior to readmission compared with weight-stable patients.

Outcome/Characteristic	Unadjusted Model			Adjusted Model		
	IRR	95% CI	*p* Value	IRR	95% CI	*p* Value
BMI	1.02	1.00, 1.04	0.043	1.02	1.00, 1.04	0.031
Age	1.01	1.00, 1.01	0.029	1.01	1.00, 1.02	0.012
CCI	1.11	1.04, 1.17	0.001	1.09	1.03, 1.16	0.004
LOS	1.04	1.01, 1.08	0.009	1.04	1.00, 1.07	0.030
ICU hours	1.51	1.37, 1.67	<0.001	1.00	0.99, 1.01	0.973

IRR = Incidence Rate Ratio.

**Table 4:** Multilevel regression model comparing clinical outcomes of patients with >5% weight loss with weight-stable patients in their readmission.

Outcome	Unadjusted Model			Adjusted Model		
	OR/IRR	95% CI	*p* Value	OR/IRR	95% CI	*p* Value
LOS	1.25	1.20, 1.30	<0.001	1.17	1.12, 1.22	<0.001
RSI	2.39	2.29, 2.49	<0.001	2.37	2.27, 2.47	<0.001
One-year mortality	2.02	1.40, 2.90	<0.001	1.50	0.99, 2.26	0.055
ICU hours	3.20	3.03, 3.37	<0.001	2.80	2.65, 2.96	<0.001
ICU admission	1.31	0.75, 2.28	0.343	1.11	0.62, 2.01	0.725
RR30	0.95	0.70, 1.28	0.725	0.96	0.70, 1.31	0.801

OR/IRR = Odds Ratio/Incidence Rate Ratio, (OR used for ICU admission, one-year mortality, RR30; IRR for LOS, RSI and ICU hours).

## Data Availability

The data presented in this study are available on request from the corresponding author only after permission is granted by the ethics committee.

## Supplementary Materials

The following supporting information can be downloaded at: https://www.mdpi.com/article/10.3390/jcm12093074/s1, Figure S1: Histograms–test for normal distribution.
