# Supplementary figures and images for: The Impact of Weight Loss Prior to Hospital Readmission
Source: J Clin Med. 2023 Apr 24;12(9):3074. doi: 10.3390/jcm12093074 (PMC10179303; doi:10.3390/jcm12093074)

Figure S1. Histograms – Test for normal distributions

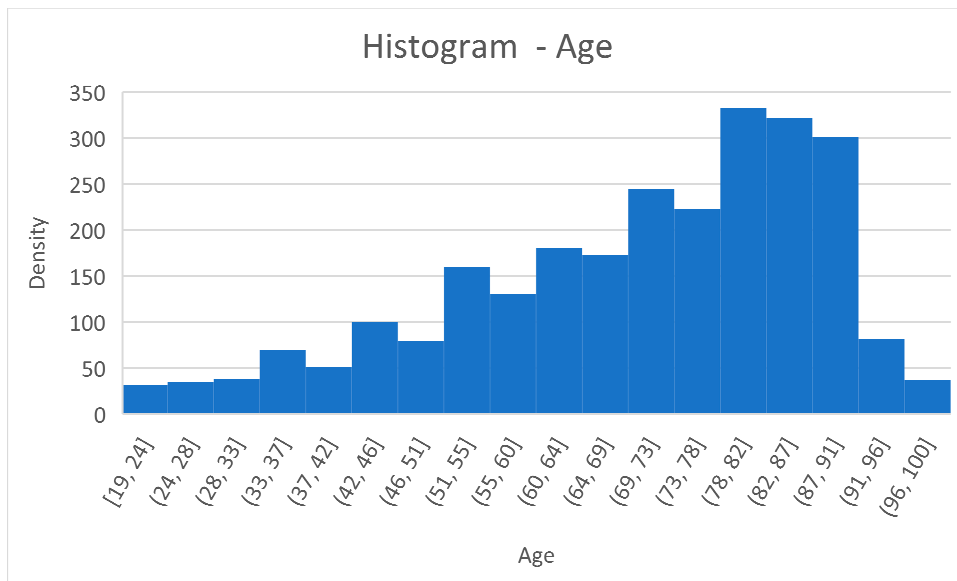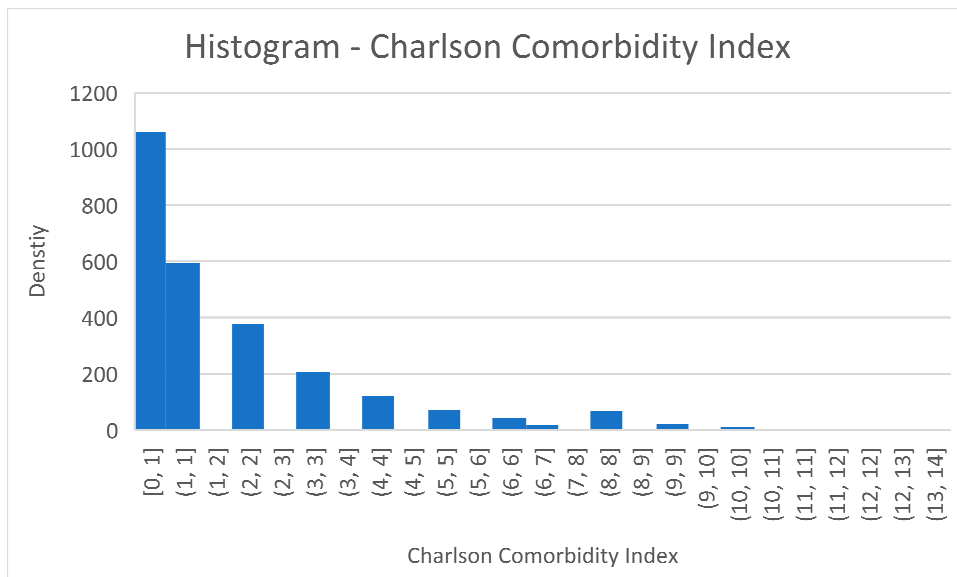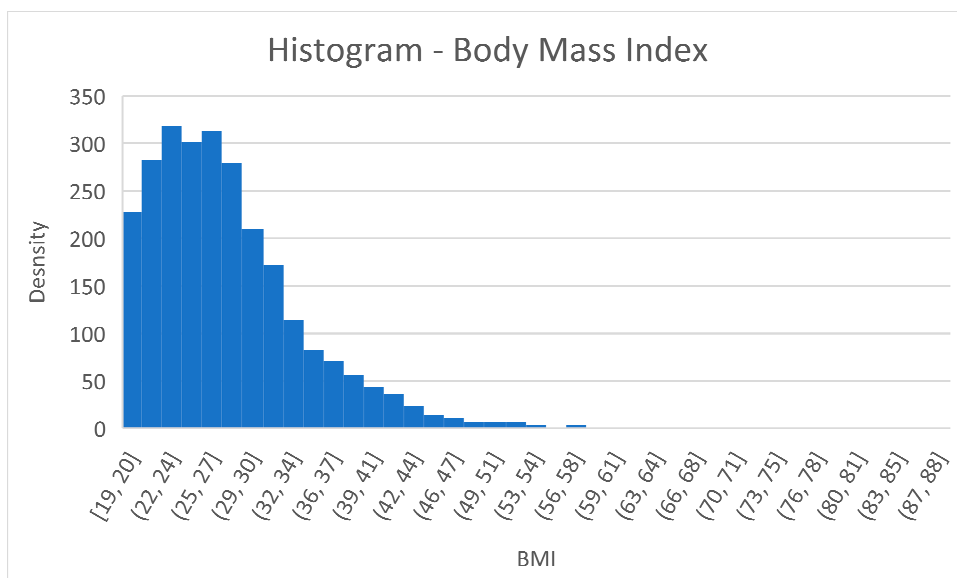

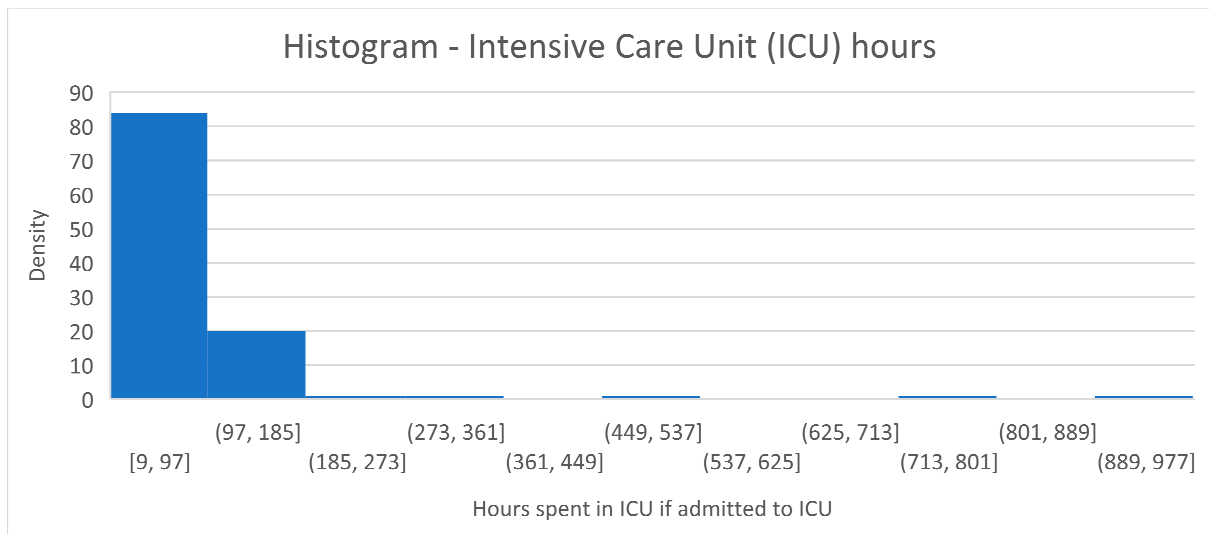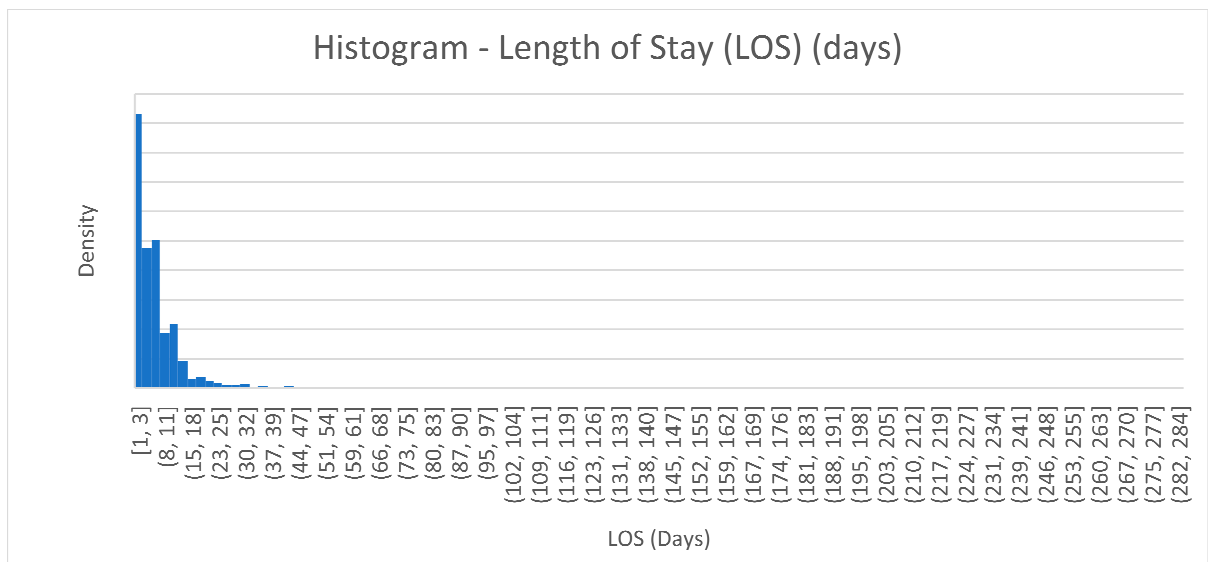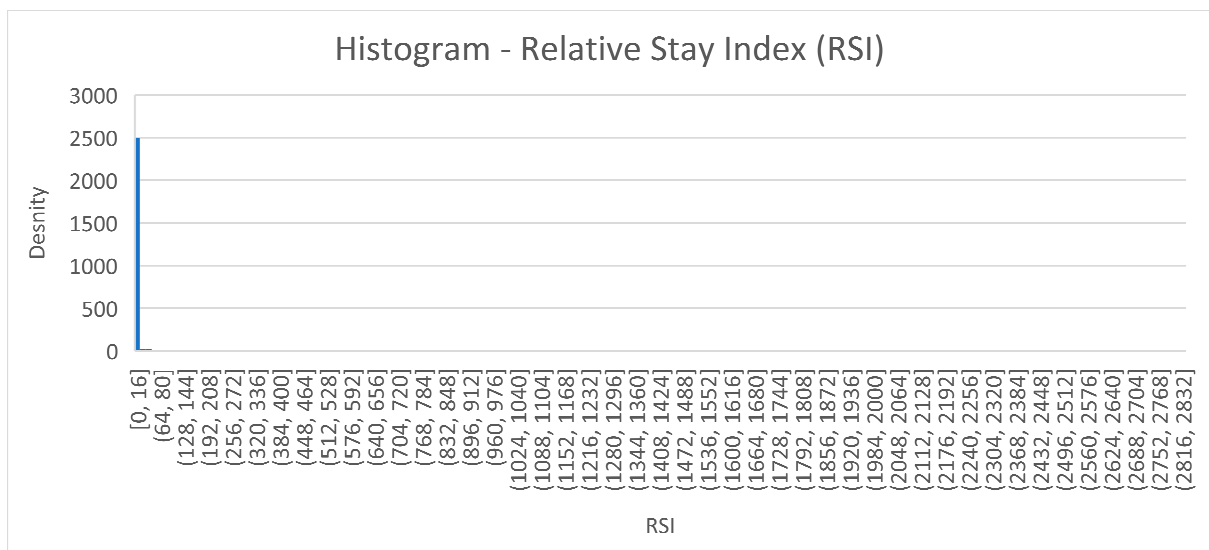

Supplement: Supplementary file 1 [file jcm-12-03074-s001.zip › jcm-2304472-supplementary.pdf]
